# Evaluation of the erosive capacity of children’s beverages on primary teeth enamel: An *in vitro* study

**DOI:** 10.4317/jced.54546

**Published:** 2018-04-01

**Authors:** Daniel-Gheur Tocolini, Mariana Dalledone, João-Armando Brancher, Juliana-Feltrin de Souza, Carla-Castiglia Gonzaga

**Affiliations:** 1 PhD student, Graduate Program in Dentistry, Universidade Positivo, Curitiba, Brazil; 2 Professor, Graduate Program in Dentistry, Universidade Positivo, Curitiba, Brazil; 3 Professor, Department of Stomatology, Federal University of Paraná Curitiba, Brazil

## Abstract

**Background:**

The consumption of acidified beverages, associated with lower mineralization of primary enamel, is the ideal combination for the development and progression of dental erosion. The objective of this study is to analyze the erosive capacity and the surface roughness of primary teeth enamel after exposure to three different brands of grape juices.

**Material and Methods:**

Forty enamel blocks of primary teeth were obtained, attened and polished. They were submitted to initial surface roughness analysis (baseline), and randomly assigned into four groups (n = 10): NAT: natural grape juice (Campo Largo); IND: industrialized grape juice (Dell Vale Kapo); SOY: soy-based grape juice (Ades); and CONT (control): artificial saliva. Blocks were immersed for 2 min, 3 times per day, for 9 days. During the whole time of the experiment, the enamel blocks were stored in artificial saliva. After the 9 days, the roughness parameters were determined again. The beverages were analyzed for pH and titratable acidity. Data were statistically analyzed (α = 0.05).

**Results:**

The surface roughness did not differ significantly among groups (*p* > 0.05). However, after the immersion in the different grape juices, the surface roughness values increased significantly (*p*< 0.05). The pH values were weakly correlated to acidity values; NAT showed the highest titratable acidity values than the other juices (*p*<0.05).

**Conclusions:**

Although the surface roughness values of the experimental groups did not differ from the control group, there was a difference in initial and final roughness in all groups. Grape juices, especially natural, may have an erosive capacity, changing the surface roughness of primary dental enamel.

** Key words:**Dental erosion, beverages, enamel, roughness.

## Introduction

In recent decades, because of public health measures, there is a clear decline in tooth decay rates in populations of different regions of the planet; however, clinicians began to worry about other changes that affect the hard dental tissue, such as enamel hypomineralization, abrasion and tooth erosion (1,2). Naturally, there is a physiological enamel wear throughout life, but the pathologic tooth wear became prevalent in people of all ages and is especially worrying in primary dentition (3).

The loss of the mineral teeth material is caused by a combination of factors, but regarding dental erosion, the main cause is the acid attack on the tooth surface by acids of non-bacterial origin but by intrinsic acids, from diet or even aspirated or inhaled (4,5).

Among the intrinsic acids, the stomach hydrochloric acid, from vomiting or gastroesophageal reflux, and those contained in beverages, are the most important. Studies on the effects of beverages on tooth structure are not recent (6). A meta-analysis study showed a significant association between dental erosion and consumption of acidic drinks (7). Despite the saliva present in the oral cavity mitigates the deleterious effects of acidic beverages, regular consumption of such beverages markedly decreases the saliva buffer capacity, which results in demineralization of tooth structure (8,9).

With regard to children, extra care must be taken because the consumption of acidified beverages, associated with lower mineralization of primary enamel, is the ideal combination for the development and progression of dental erosion. Because the process is slow and progressive, early clinical diagnosis and identification of etiologic factors involved is the key to prevention.

Thus, the aim of this study was to evaluate the surface roughness of primary teeth enamel after exposure to three different brands of grape juices. In addition, pH and titratable acidity of beverages were evaluated.

## Material and Methods

This study was approved by the Institutional Review Board and used 40 donated primary teeth. The inclusion criteria were primary incisors and molars having at least one of free surfaces healthy allowing the removal of a block of enamel. Teeth without coronary remaining, endodontically treated teeth, and teeth with developmental defects in tooth enamel were excluded.

Enamel blocks were prepared with dimensions of 3 mm2, were flattened and polished with sandpaper in descending granulation 400, 600, 1200. The thickness of the blocks was evaluated and standardized in all dimensions using a digital caliper.

Enamel blocks (n = 10) were then divided into 4 groups and exposed to the beverages, as follows: NAT: natural grape juice (Campo Largo Juice Company, Campo Largo, PR, Brazil); IND: industrialized grape juice (Dell Vale Kapo, The Coca Cola Company, SP Brazil); SOY: soy-based grape juice (Ades, Unilever); and CTRL (control): artificial saliva. Blocks were immersed for 2 min, 3 times per day, for 9 days. During the whole time of the experiment, the enamel blocks were stored in artificial saliva. Sample size calculation was determined based on previous similar studies (10,11). The artificial saliva contained the following composition: 150 mmol/L KCl, 1.5 mmol/L CaCl2, and 0.9 mmol/L KH2PO4 in 100 mL of distilled water (pH was adjusted to 7.0).

-Surface roughness analysis

For the roughness test, the specimens were attached to a glass slide with wax and individually analyzed with a roughness tester (SJ-210P Surftest; Mitutoyo, Kawasaki, Japan) equipped with a diamond needle with a 5-mm radius at a constant speed of 0.5 mm/s. Before the readings, the roughness tester was calibrated according to the manufacturer’s recommendations with a roughness standard. For each reading, a length of 2.5 mm was analyzed with a cutoff of 0.25 mm. All readings were performed in duplicate. Three roughness parameters were evaluated: Ra, which is rated as the arithmetic average of the surface peaks and valleys; Rz, which is the average between the peaks and valleys recorded in each of the test section; and Rq, which corresponds to the maximum distance between the highest peak and the deepest valley in the measurement path.

Data were analyzed using SPSS software (Statistical Package for the Social Sciences, version 20.0, IBM, USA). The dependent variables of the study were the roughness parameters that were initially tested for the distribution using Shapiro-Wilk normality test. To analyze differences between groups and between times (before and after), the average of each parameter (Ra, Rz and Rq) was analyzed by two-way repeated measures ANOVA and Tukey’s test. The significance level was 5%.

-pH and acidity analysis

The grape juices were analyzed for pH and titratable acidity immediately after the packages are opened. The pH analysis was performed using a digital pHmeter (PhepR+, HANNA, Hanna Instruments SP, Brazil) previously calibrated with buffer solutions of pH 4.0 and pH 7.0, according to the manufacturer’s instruction. Measurements were initially carried out before the specimens are immersed.

The acidity was determined by beverage titration with sodium hydroxide (0.1 N). The pH range during the titration was monitored with a pHmeter until the pH rose to 5.5 and 7.0 for each sample. The volume of added sodium hydroxide was recorded in mL.

The pH and acidity values to pH 5.5 and pH 7.0 of each juice (group) were analyzed for the distribution using Shapiro-Wilk normality test. Differences between groups were analyzed by one-way ANOVA and Tukey’s test. The significance level was 5%. The pH, acidity pH 5.5 and acidity pH 7.0 values were correlated by the Pearson’s correlation coefficient.

## Results

The surface roughness parameters analyzed (Ra, Rq and Rz) performed similarly, indicating statistically significant differences only for time (between baseline and final measurements) (*p* = 0.0001 for Ra, *p* = 0.0028 for Rq and *p* = 0.0002 for Rz). Groups (*p* = 0.1758 for Ra, *p* = 0.1715 for Rq and *p* = 0.1682 for Rz) and the double interaction groups*times (*p* = 0.1131 for Ra, *p* = 0.1669 for Rq and *p* = 0.1262 for Rz) were not significant. Considering the measurements time, for all roughness parameters, final means were significantly higher than baseline mean values (Ra_baseline = 0.67 µm and Ra_final = 1.10 µm; Rq_baseline = 0.89 µm and Rq_final = 1.38 µm; Rz_baseline = 3.01 µm and Rz_final = 4.92 µm). The means and standard deviations for all groups, roughness parameters and time are shown in  Table 1. It can be observed that for Ra and Rq, significant differences between baseline and final values were observed only for natural grape juice (NAT). For Rz, significant differences between baseline and final values were observed only for natural industrialized grape juice (IND).

**Table 1:**
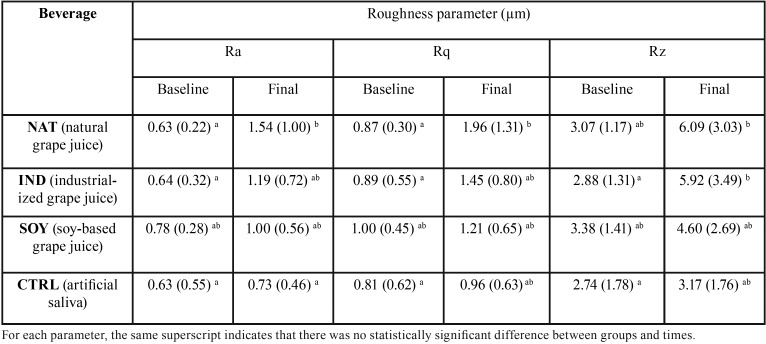
Means (SD) of the roughness parameters (µm) at baseline and after exposure to the different beverages and artificial saliva.

**Figure 1:**
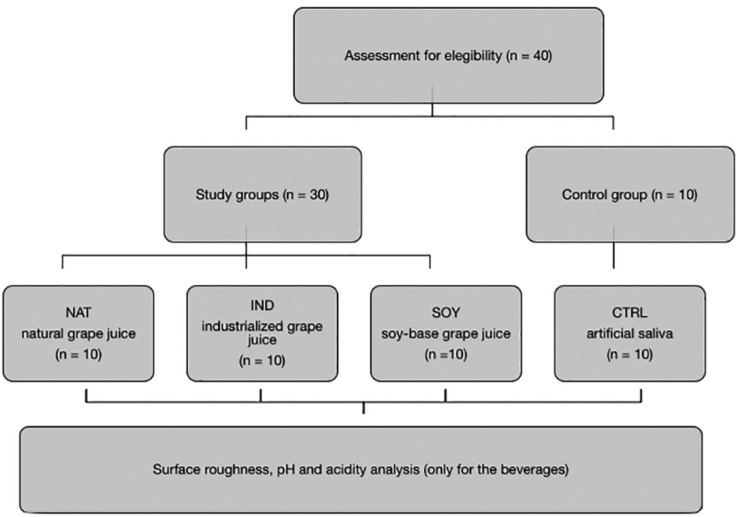
CONSORT flow diagram of the study.

The pH and acidity values for pH 5.5 and pH 7.0 in each group are shown in  Table 2. There was a statistically significant difference between groups (*p* = 0.02). The lower pH values were found in industrialized grape juice (IND) and natural grape juice (NAT), which did not differ between each other (*p* = 0.87). IND showed lower pH values than juice grape with soy milk (SOY) (*p* = 0.031) ( Table 2). The acidity values for pH 5.5 and pH 7.0 were strongly correlated by the Pearson’s correlation (r = 0.987), and weakly correlated with pH values (r = 0.33 and r = 0.66 for acidity in pH 5.5 and pH and acidity pH 7.0, respectively). The natural grape juice (NAT) showed the highest acidity values, differing from IND and SOY (*p* < 0.001). While the IND and SOY values did not differ between each other (*p* = 0.697), (Fig. 1).

**Table 2:**
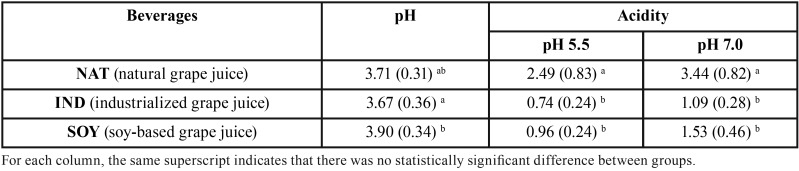
Means (SD) of pH and acidity values for pH 5.5 and pH 7.0 for the different beverages.

## Discussion

In this study, the surface roughness of primary teeth after exposure to different beverages was evaluated. In order to mimic what happens in the oral cavity, the teeth were stored in artificial saliva with appropriate concentrations of minerals, after exposure to acidic beverages. Such beverages have characteristics inherent to them and that are decisive for their erosive potential. Among these features the pH is extremely important. It is known that pH refers to the concentration of free hydrogen ions in solution, therefore it shows the actual availability of these ions to interact with the tooth surface (12). In this study the evaluation of beverages showed that their pH was less than 4.0, with average value of 3.7, being considered potentially erosive.

When pH decreases, the solubility of hydroxyapatite, both in enamel and dentine, increases. However, this is not the only factor in the cause of mineral dissolution by erosion. In this study, pH values are weakly correlated to the acidity values. This could be explained analyzing other variables such as amount of titratable acids, type of acids, buffer capacity and chelating properties. Naturally, grape contains tartaric and maleic acids. Other acids can be added in industrialized beverages, acting as anti-oxidants, such as ascorbic acid and pH regulators, such as citric acid. The presence of these components may be related to the time of development of dental erosion and allergic reactions (13).

The presence of phosphate, calcium and fluoride in the composition of beverages should also be considered (12). Undissociated organic acids should also be taken into account (7,15,16). The measurement of these in beverages or any solutions is performed by means of titration, a technique calls for the use of a base, NaOH, to titrate the acidic solution. The greater the amount of NaOH added to the analyzed sample, the higher is its acidity.

In the present study, the natural grape juice showed the highest acidity values, as it required a statistically higher amount of NaOH so that the pH rose to 5.5 and 7.0 (*p* < 0.001) compared to the other two groups. However, the obtained results did not correlate to the acidity observed in this juice. Once there are differences between the erosive potential of beverages and their pH and acidity, the enamel surface analysis is very important to determine the action of the juices on its surface (17-20). In this study, for the three roughness parameters evaluated, no statistically significant differences among the groups (NAT, IND, SOY and CTRL). The low pH as well as the low calcium and fluoride ion concentration indicates higher erosive potential (21). A commonly investigated modification has been the use of additives, mostly salts containing calcium and/or phosphate ions. They act based on the common ion effect, where the driving force for dental surface dissolution can be decreased by the saturated state of the beverage with respect to the calcium and phosphate ions (20).

In this study the natural grape juice (NAT) showed the highest acidity values, statistically differing from IND and SOY (*p* < 0.001), while IND and SOY values did not differ between them (*p* = 0.697). However, other studies report that the whole juice showed the highest titratable acidity, significantly differing from the other flavors (22-25).

An important point to be considered is the exposure time of the enamel samples in the juice. Analyzing the erosive effect of different grape juices on the surface roughness, it was noted that only after 15 days of exposure the grape juices caused an increase in surface roughness (14,26). In this study, even without statistical difference, it was observed that a 9-day exposure to beverages had an increase in surface roughness of specimens evaluated and it is assumed that, if the exposure time were greater, the difference would be significant.

Considering that, parents should be aware about healthy life habits of their children. There is an increase in consumption of natural juices, which replace soft drinks; however, data from previous studies and this study showed that these beverages cause tooth enamel demineralization and should be consumed with caution (12,27,28). Thus, although tooth erosion is multifactorial, the frequent consumption of foods with low pH and high acid content can be a determining factor in its evolution.

## Conclusions

It can be concluded that all grape juices tested influenced in the roughness parameters of primary enamel, even if they did not differ from the control group. Grape juices, especially natural, may have erosive capacity, changing the surface roughness of primary dental enamel. pH had a weak correlation with the acidity values and the juice with highest acidity (natural grape juice) showed greater erosive.
